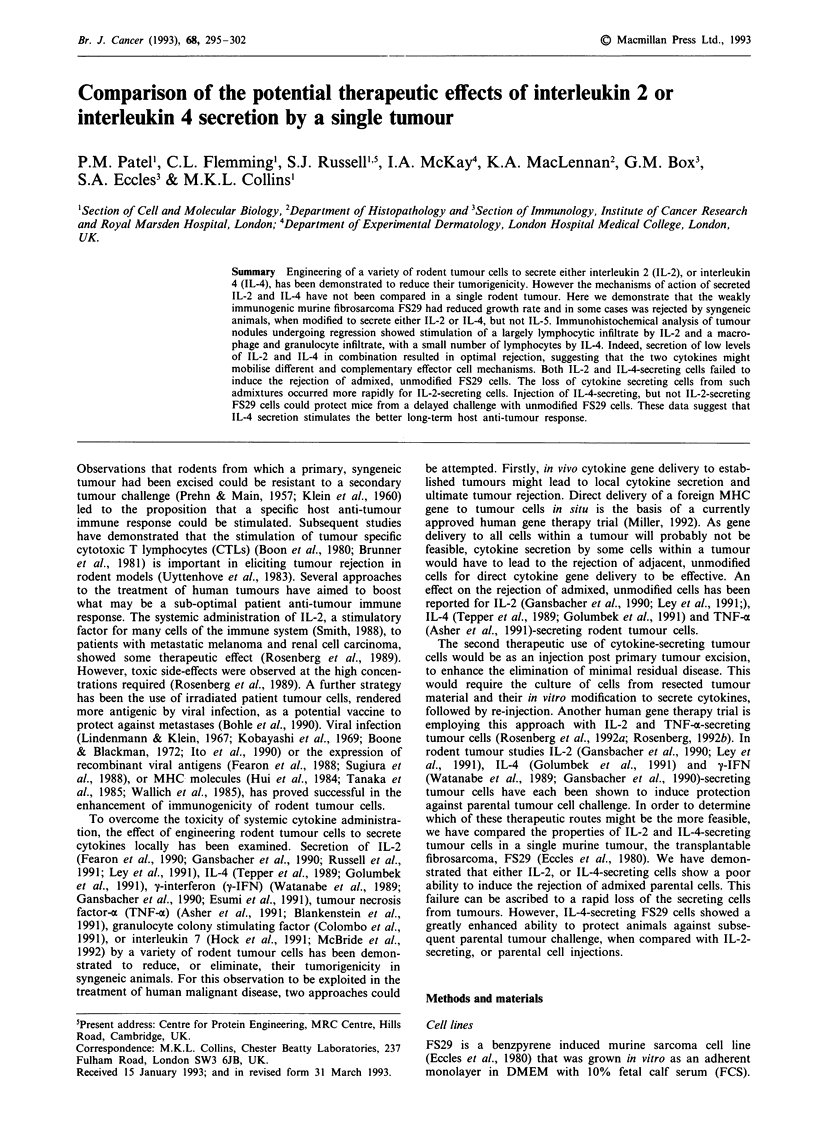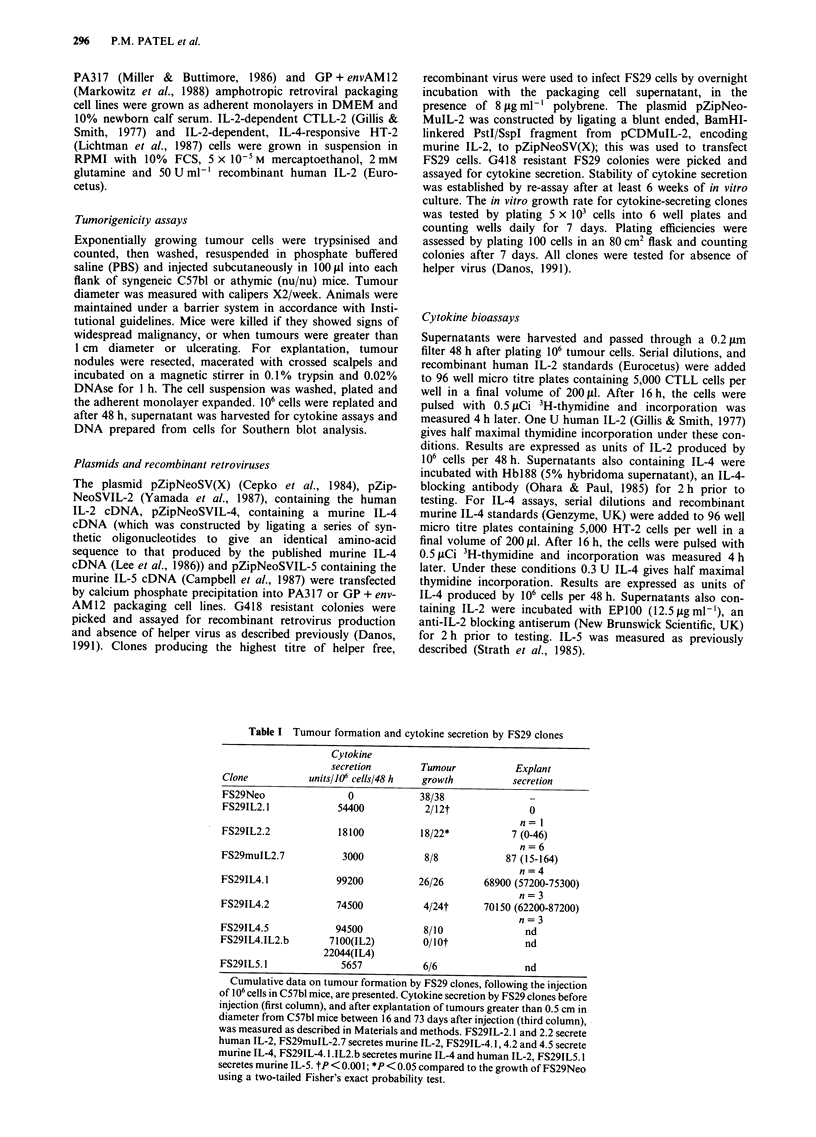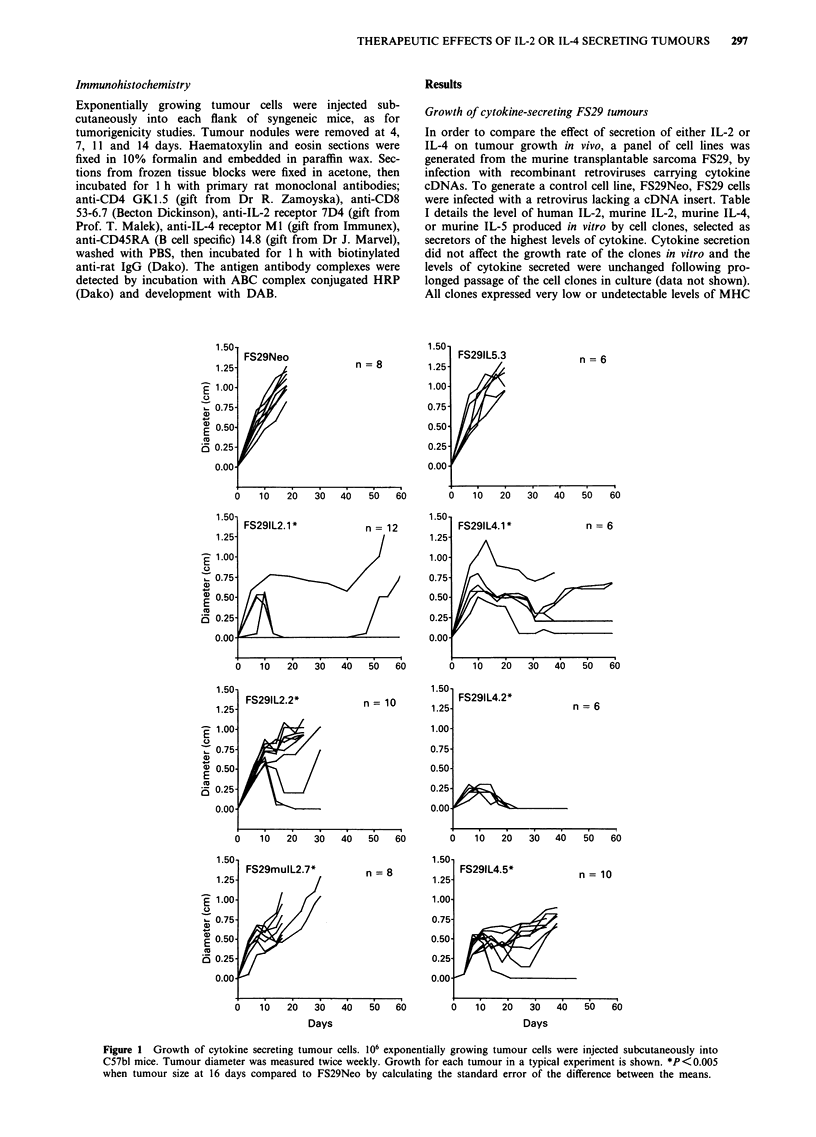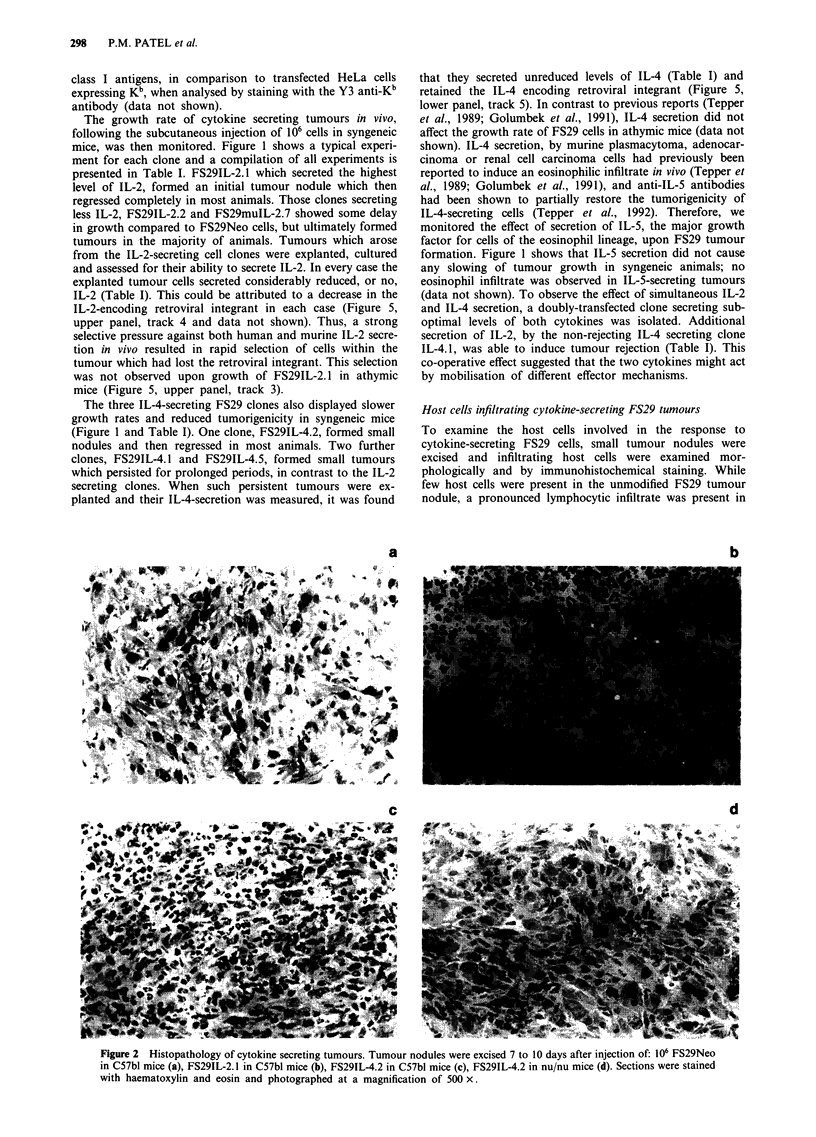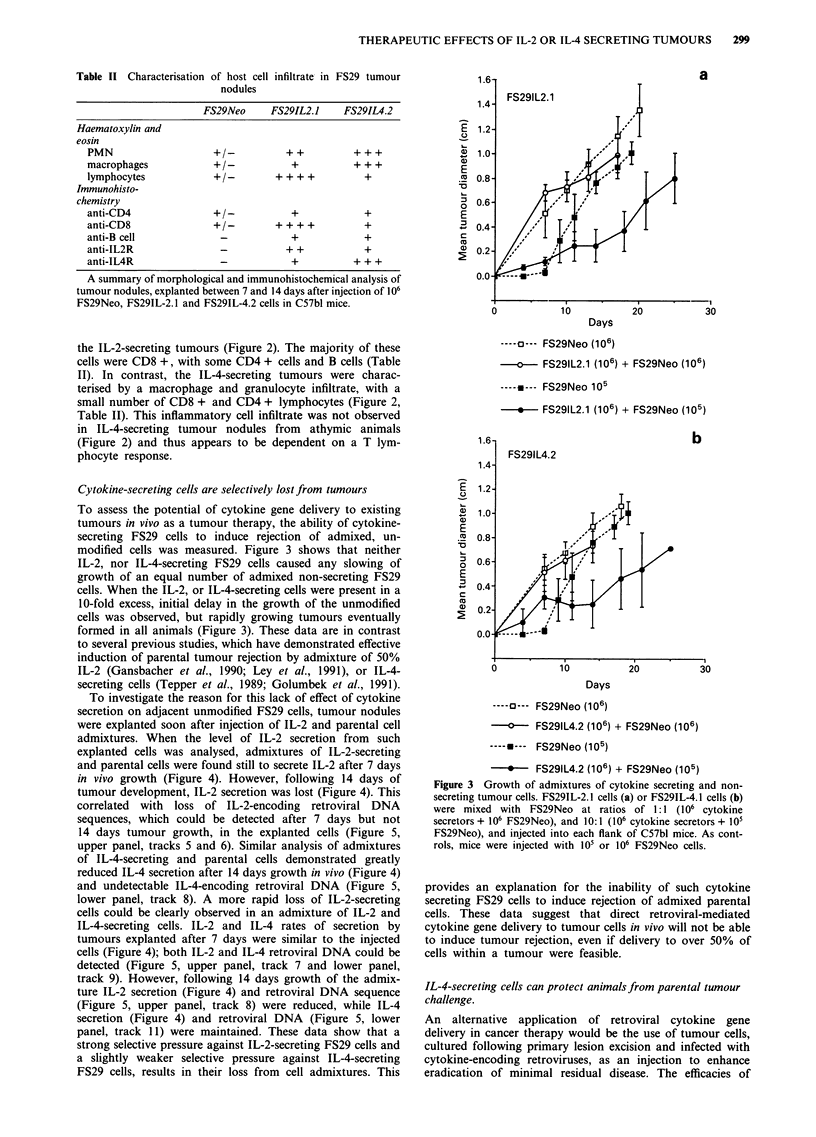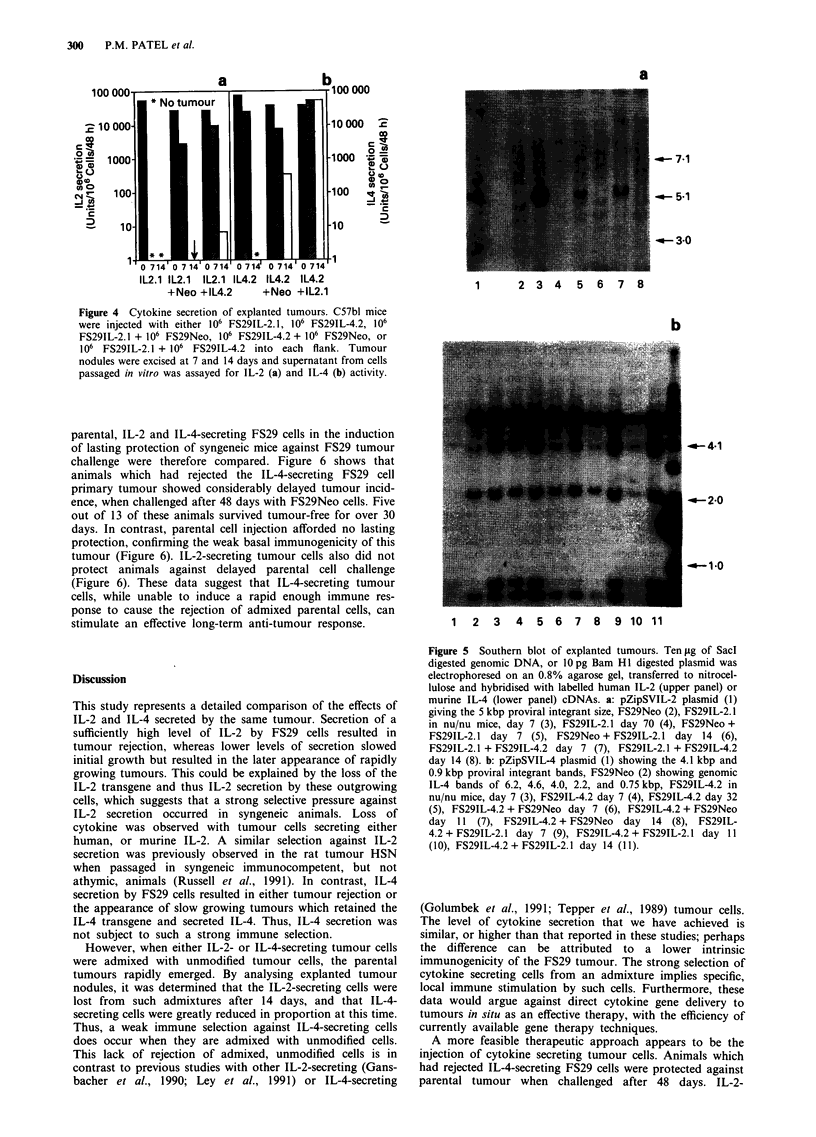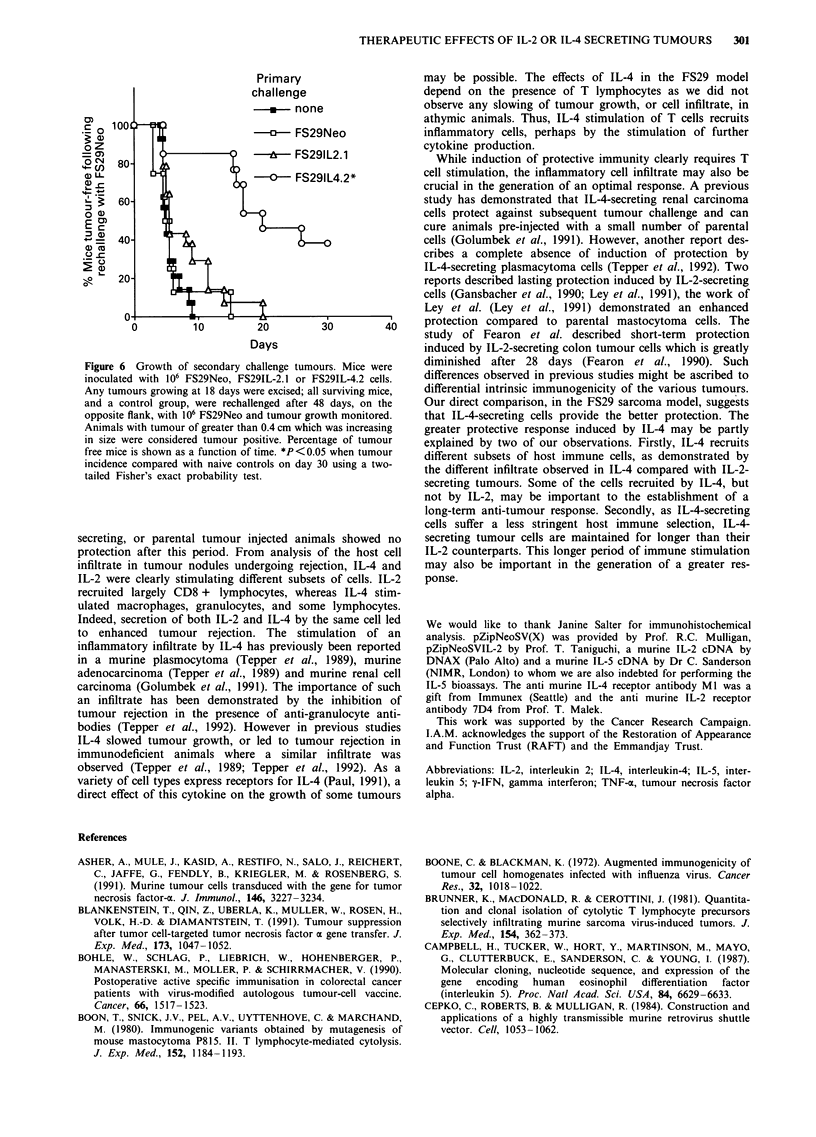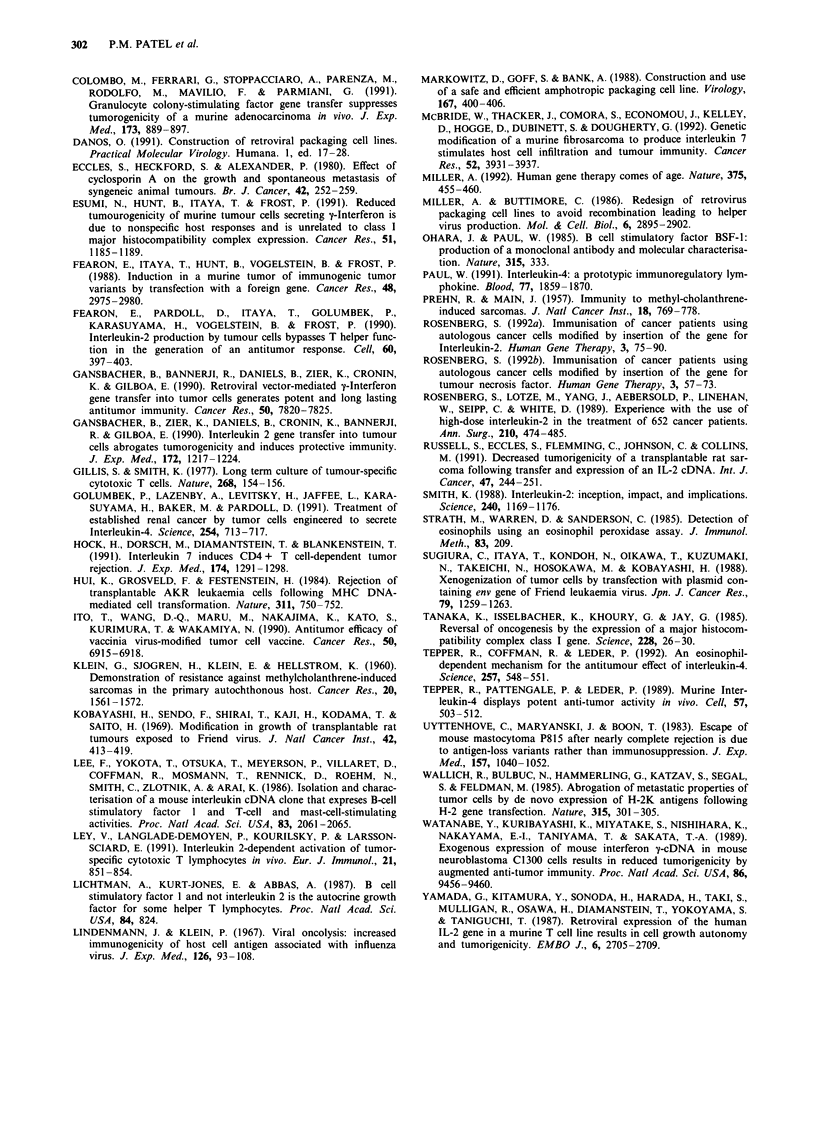# Comparison of the potential therapeutic effects of interleukin 2 or interleukin 4 secretion by a single tumour.

**DOI:** 10.1038/bjc.1993.331

**Published:** 1993-08

**Authors:** P. M. Patel, C. L. Flemming, S. J. Russell, I. A. McKay, K. A. MacLennan, G. M. Box, S. A. Eccles, M. K. Collins

**Affiliations:** Section of Cell and Molecular Biology, London Hospital Medical College, UK.

## Abstract

**Images:**


Br. J. Cancer (1993), 68, 295–302 © Macmillan Press Ltd., 1993

### Comparison of the potential therapeutic effects of interleukin 2 or interleukin 4 secretion by a single tumour

P.M. Patel[1], C.L. Flemming[1], S.J. Russell[1,5], I.A. McKay[4], K.A. MacLennan[2], G.M. Box[3], S.A. Eccles[3] & M.K.L. Collins[1]

[1]*Section of Cell and Molecular Biology,* [2]*Department of Histopathology and* [3]*Section of Immunology, Institute of Cancer Research and Royal Marsden Hospital, London;* [4]*Department of Experimental Dermatology, London Hospital Medical College, London, UK.*

**Summary** Engineering of a variety of rodent tumour cells to secrete either interleukin 2 (IL-2), or interleukin 4 (IL-4), has been demonstrated to reduce their tumorigenicity. However the mechanisms of action of secreted IL-2 and IL-4 have not been compared in a single rodent tumour. Here we demonstrate that the weakly immunogenic murine fibrosarcoma FS29 had reduced growth rate and in some cases was rejected by syngeneic animals, when modified to secrete either IL-2 or IL-4, but not IL-5. Immunohistochemical analysis of tumour nodules undergoing regression showed stimulation of a largely lymphocytic infiltrate by IL-2 and a macrophage and granulocyte infiltrate, with a small number of lymphocytes by IL-4. Indeed, secretion of low levels of IL-2 and IL-4 in combination resulted in optimal rejection, suggesting that the two cytokines might mobilise different and complementary effector cell mechanisms. Both IL-2 and IL-4-secreting cells failed to induce the rejection of admixed, unmodified FS29 cells. The loss of cytokine secreting cells from such admixtures occurred more rapidly for IL-2-secreting cells. Injection of IL-4-secreting, but not IL-2-secreting FS29 cells could protect mice from a delayed challenge with unmodified FS29 cells. These data suggest that IL-4 secretion stimulates the better long-term host anti-tumour response.

Observations that rodents from which a primary, syngeneic tumour had been excised could be resistant to a secondary tumour challenge (Prehn & Main, 1957; Klein *et al.*, 1960) led to the proposition that a specific host anti-tumour immune response could be stimulated. Subsequent studies have demonstrated that the stimulation of tumour specific cytotoxic T lymphocytes (CTLs) (Boon *et al.*, 1980; Brunner *et al.*, 1981) is important in eliciting tumour rejection in rodent models (Uyttenhove *et al.*, 1983). Several approaches to the treatment of human tumours have aimed to boost what may be a sub-optimal patient anti-tumour immune response. The systemic administration of IL-2, a stimulatory factor for many cells of the immune system (Smith, 1988), to patients with metastatic melanoma and renal cell carcinoma, showed some therapeutic effect (Rosenberg *et al.*, 1989). However, toxic side-effects were observed at the high concentrations required (Rosenberg *et al.*, 1989). A further strategy has been the use of irradiated patient tumour cells, rendered more antigenic by viral infection, as a potential vaccine to protect against metastases (Bohle *et al.*, 1990). Viral infection (Lindenmann & Klein, 1967; Kobayashi *et al.*, 1969; Boone & Blackman, 1972; Ito *et al.*, 1990) or the expression of recombinant viral antigens (Fearon *et al.*, 1988; Sugiura *et al.*, 1988), or MHC molecules (Hui *et al.*, 1984; Tanaka *et al.*, 1985; Wallich *et al.*, 1985), has proved successful in the enhancement of immunogenicity of rodent tumour cells.

To overcome the toxicity of systemic cytokine administration, the effect of engineering rodent tumour cells to secrete cytokines locally has been examined. Secretion of IL-2 (Fearon *et al.*, 1990; Gansbacher *et al.*, 1990; Russell *et al.*, 1991; Ley *et al.*, 1991), IL-4 (Tepper *et al.*, 1989; Golumbek *et al.*, 1991), γ-interferon (γ-IFN) (Watanabe *et al.*, 1989; Gansbacher *et al.*, 1990; Esumi *et al.*, 1991), tumour necrosis factor-α (TNF-α) (Asher *et al.*, 1991; Blankenstein *et al.*, 1991), granulocyte colony stimulating factor (Colombo *et al.*, 1991), or interleukin 7 (Hock *et al.*, 1991; McBride *et al.*, 1992) by a variety of rodent tumour cells has been demonstrated to reduce, or eliminate, their tumorigenicity in syngeneic animals. For this observation to be exploited in the treatment of human malignant disease, two approaches could be attempted. Firstly, *in vivo* cytokine gene delivery to established tumours might lead to local cytokine secretion and ultimate tumour rejection. Direct delivery of a foreign MHC gene to tumour cells *in situ* is the basis of a currently approved human gene therapy trial (Miller, 1992). As gene delivery to all cells within a tumour will probably not be feasible, cytokine secretion by some cells within a tumour would have to lead to the rejection of adjacent, unmodified cells for direct cytokine gene delivery to be effective. An effect on the rejection of admixed, unmodified cells has been reported for IL-2 (Gansbacher *et al.*, 1990; Ley *et al.*, 1991;), IL-4 (Tepper *et al.*, 1989; Golumbek *et al.*, 1991) and TNF-α (Asher *et al.*, 1991)-secreting rodent tumour cells.

The second therapeutic use of cytokine-secreting tumour cells would be as an injection post primary tumour excision, to enhance the elimination of minimal residual disease. This would require the culture of cells from resected tumour material and their *in vitro* modification to secrete cytokines, followed by re-injection. Another human gene therapy trial is employing this approach with IL-2 and TNF-α-secreting tumour cells (Rosenberg *et al.*, 1992*a*; Rosenberg, 1992*b*). In rodent tumour studies IL-2 (Gansbacher *et al.*, 1990; Ley *et al.*, 1991), IL-4 (Golumbek *et al.*, 1991) and γ-IFN (Watanabe *et al.*, 1989; Gansbacher *et al.*, 1990)-secreting tumour cells have each been shown to induce protection against parental tumour cell challenge. In order to determine which of these therapeutic routes might be the more feasible, we have compared the properties of IL-2 and IL-4-secreting tumour cells in a single murine tumour, the transplantable fibrosarcoma, FS29 (Eccles *et al.*, 1980). We have demonstrated that either IL-2, or IL-4-secreting cells show a poor ability to induce the rejection of admixed parental cells. This failure can be ascribed to a rapid loss of the secreting cells from tumours. However, IL-4-secreting FS29 cells showed a greatly enhanced ability to protect animals against subsequent parental tumour challenge, when compared with IL-2-secreting, or parental cell injections.

### Methods and materials

### *Cell lines*

FS29 is a benzpyrene induced murine sarcoma cell line (Eccles *et al.*, 1980) that was grown *in vitro* as an adherent monolayer in DMEM with 10% fetal calf serum (FCS).

[5]Present address: Centre for Protein Engineering, MRC Centre, Hills Road, Cambridge, UK.
Correspondence: M.K.L. Collins, Chester Beatty Laboratories, 237 Fulham Road, London SW3 6JB, UK.
Received 15 January 1993; and in revised form 31 March 1993.

PA317 (Miller & Buttimore, 1986) and GP + *env*AM12 (Markowitz *et al.*, 1988) amphotropic retroviral packaging cell lines were grown as adherent monolayers in DMEM and 10% newborn calf serum. IL-2-dependent CTLL-2 (Gillis & Smith, 1977) and IL-2-dependent, IL-4-responsive HT-2 (Lichtman *et al.*, 1987) cells were grown in suspension in RPMI with 10% FCS, $5 \times 10^{-5}$ M mercaptoethanol, 2 mM glutamine and 50 U $ml^{-1}$ recombinant human IL-2 (Eurocetus).

*Tumorigenicity assays*

Exponentially growing tumour cells were trypsinised and counted, then washed, resuspended in phosphate buffered saline (PBS) and injected subcutaneously in 100 $\mu$l into each flank of syngeneic C57bl or athymic (nu/nu) mice. Tumour diameter was measured with calipers X2/week. Animals were maintained under a barrier system in accordance with Institutional guidelines. Mice were killed if they showed signs of widespread malignancy, or when tumours were greater than 1 cm diameter or ulcerating. For explantation, tumour nodules were resected, macerated with crossed scalpels and incubated on a magnetic stirrer in 0.1% trypsin and 0.02% DNAse for 1 h. The cell suspension was washed, plated and the adherent monolayer expanded. $10^6$ cells were replated and after 48 h, supernatant was harvested for cytokine assays and DNA prepared from cells for Southern blot analysis.

*Plasmids and recombinant retroviruses*

The plasmid pZipNeoSV(X) (Cepko *et al.*, 1984), pZipNeoSVIL-2 (Yamada *et al.*, 1987), containing the human IL-2 cDNA, pZipNeoSVIL-4, containing a murine IL-4 cDNA (which was constructed by ligating a series of synthetic oligonucleotides to give an identical amino-acid sequence to that produced by the published murine IL-4 cDNA (Lee *et al.*, 1986)) and pZipNeoSVIL-5 containing the murine IL-5 cDNA (Campbell *et al.*, 1987) were transfected by calcium phosphate precipitation into PA317 or GP + *env*-AM12 packaging cell lines. G418 resistant colonies were picked and assayed for recombinant retrovirus production and absence of helper virus as described previously (Danos, 1991). Clones producing the highest titre of helper free, recombinant virus were used to infect FS29 cells by overnight incubation with the packaging cell supernatant, in the presence of 8 $\mu$g $ml^{-1}$ polybrene. The plasmid pZipNeo-MuIL-2 was constructed by ligating a blunt ended, BamHI-linkered PstI/SspI fragment from pCDMuIL-2, encoding murine IL-2, to pZipNeoSV(X); this was used to transfect FS29 cells. G418 resistant FS29 colonies were picked and assayed for cytokine secretion. Stability of cytokine secretion was established by re-assay after at least 6 weeks of *in vitro* culture. The *in vitro* growth rate for cytokine-secreting clones was tested by plating $5 \times 10^3$ cells into 6 well plates and counting wells daily for 7 days. Plating efficiencies were assessed by plating 100 cells in an 80 $cm^2$ flask and counting colonies after 7 days. All clones were tested for absence of helper virus (Danos, 1991).

*Cytokine bioassays*

Supernatants were harvested and passed through a 0.2 $\mu$m filter 48 h after plating $10^6$ tumour cells. Serial dilutions, and recombinant human IL-2 standards (Eurocetus) were added to 96 well micro titre plates containing 5,000 CTLL cells per well in a final volume of 200 $\mu$l. After 16 h, the cells were pulsed with 0.5 $\mu$Ci $^3$H-thymidine and incorporation was measured 4 h later. One U human IL-2 (Gillis & Smith, 1977) gives half maximal thymidine incorporation under these conditions. Results are expressed as units of IL-2 produced by $10^6$ cells per 48 h. Supernatants also containing IL-4 were incubated with Hb188 (5% hybridoma supernatant), an IL-4-blocking antibody (Ohara & Paul, 1985) for 2 h prior to testing. For IL-4 assays, serial dilutions and recombinant murine IL-4 standards (Genzyme, UK) were added to 96 well micro titre plates containing 5,000 HT-2 cells per well in a final volume of 200 $\mu$l. After 16 h, the cells were pulsed with 0.5 $\mu$Ci $^3$H-thymidine and incorporation was measured 4 h later. Under these conditions 0.3 U IL-4 gives half maximal thymidine incorporation. Results are expressed as units of IL-4 produced by $10^6$ cells per 48 h. Supernatants also containing IL-2 were incubated with EP100 (12.5 $\mu$g $ml^{-1}$), an anti-IL-2 blocking antiserum (New Brunswick Scientific, UK) for 2 h prior to testing. IL-5 was measured as previously described (Strath *et al.*, 1985).

**Table I** Tumour formation and cytokine secretion by FS29 clones

| Clone | Cytokine secretion units/$10^6$ cells/48 h | Tumour growth | Explant secretion |
|---|---|---|---|
| FS29Neo | 0 | 38/38 | – |
| FS29IL2.1 | 54400 | 2/12† | 0<br>n = 1 |
| FS29IL2.2 | 18100 | 18/22* | 7 (0-46)<br>n = 6 |
| FS29muIL2.7 | 3000 | 8/8 | 87 (15-164)<br>n = 4 |
| FS29IL4.1 | 99200 | 26/26 | 68900 (57200-75300)<br>n = 3 |
| FS29IL4.2 | 74500 | 4/24† | 70150 (62200-87200)<br>n = 3 |
| FS29IL4.5 | 94500 | 8/10 | nd |
| FS29IL4.IL2.b | 7100(IL2)<br>22044(IL4) | 0/10† | nd |
| FS29IL5.1 | 5657 | 6/6 | nd |

Cumulative data on tumour formation by FS29 clones, following the injection of $10^6$ cells in C57bl mice, are presented. Cytokine secretion by FS29 clones before injection (first column), and after explantation of tumours greater than 0.5 cm in diameter from C57bl mice between 16 and 73 days after injection (third column), was measured as described in Materials and methods. FS29IL-2.1 and 2.2 secrete human IL-2, FS29muIL-2.7 secretes murine IL-2, FS29IL-4.1, 4.2 and 4.5 secrete murine IL-4, FS29IL-4.1.IL2.b secretes murine IL-4 and human IL-2, FS29IL5.1 secretes murine IL-5. †$P < 0.001$; *$P < 0.05$ compared to the growth of FS29Neo using a two-tailed Fisher's exact probability test.

### *Immunohistochemistry*

Exponentially growing tumour cells were injected subcutaneously into each flank of syngeneic mice, as for tumorigenicity studies. Tumour nodules were removed at 4, 7, 11 and 14 days. Haematoxylin and eosin sections were fixed in 10% formalin and embedded in paraffin wax. Sections from frozen tissue blocks were fixed in acetone, then incubated for 1 h with primary rat monoclonal antibodies; anti-CD4 GK1.5 (gift from Dr R. Zamoyska), anti-CD8 53-6.7 (Becton Dickinson), anti-IL-2 receptor 7D4 (gift from Prof. T. Malek), anti-IL-4 receptor M1 (gift from Immunex), anti-CD45RA (B cell specific) 14.8 (gift from Dr J. Marvel), washed with PBS, then incubated for 1 h with biotinylated anti-rat IgG (Dako). The antigen antibody complexes were detected by incubation with ABC complex conjugated HRP (Dako) and development with DAB.

### Results

### *Growth of cytokine-secreting FS29 tumours*

In order to compare the effect of secretion of either IL-2 or IL-4 on tumour growth *in vivo*, a panel of cell lines was generated from the murine transplantable sarcoma FS29, by infection with recombinant retroviruses carrying cytokine cDNAs. To generate a control cell line, FS29Neo, FS29 cells were infected with a retrovirus lacking a cDNA insert. Table I details the level of human IL-2, murine IL-2, murine IL-4, or murine IL-5 produced *in vitro* by cell clones, selected as secretors of the highest levels of cytokine. Cytokine secretion did not affect the growth rate of the clones *in vitro* and the levels of cytokine secreted were unchanged following prolonged passage of the cell clones in culture (data not shown). All clones expressed very low or undetectable levels of MHC

**Figure 1** Growth of cytokine secreting tumour cells. $10^6$ exponentially growing tumour cells were injected subcutaneously into C57bl mice. Tumour diameter was measured twice weekly. Growth for each tumour in a typical experiment is shown. *$P < 0.005$ when tumour size at 16 days compared to FS29Neo by calculating the standard error of the difference between the means.

class I antigens, in comparison to transfected HeLa cells expressing $K^b$, when analysed by staining with the Y3 anti-$K^b$ antibody (data not shown).

The growth rate of cytokine secreting tumours *in vivo*, following the subcutaneous injection of $10^6$ cells in syngeneic mice, was then monitored. Figure 1 shows a typical experiment for each clone and a compilation of all experiments is presented in Table I. FS29IL-2.1 which secreted the highest level of IL-2, formed an initial tumour nodule which then regressed completely in most animals. Those clones secreting less IL-2, FS29IL-2.2 and FS29muIL-2.7 showed some delay in growth compared to FS29Neo cells, but ultimately formed tumours in the majority of animals. Tumours which arose from the IL-2-secreting cell clones were explanted, cultured and assessed for their ability to secrete IL-2. In every case the explanted tumour cells secreted considerably reduced, or no, IL-2 (Table I). This could be attributed to a decrease in the IL-2-encoding retroviral integrant in each case (Figure 5, upper panel, track 4 and data not shown). Thus, a strong selective pressure against both human and murine IL-2 secretion *in vivo* resulted in rapid selection of cells within the tumour which had lost the retroviral integrant. This selection was not observed upon growth of FS29IL-2.1 in athymic mice (Figure 5, upper panel, track 3).

The three IL-4-secreting FS29 clones also displayed slower growth rates and reduced tumorigenicity in syngeneic mice (Figure 1 and Table I). One clone, FS29IL-4.2, formed small nodules and then regressed in most animals. Two further clones, FS29IL-4.1 and FS29IL-4.5, formed small tumours which persisted for prolonged periods, in contrast to the IL-2 secreting clones. When such persistent tumours were explanted and their IL-4-secretion was measured, it was found that they secreted unreduced levels of IL-4 (Table I) and retained the IL-4 encoding retroviral integrant (Figure 5, lower panel, track 5). In contrast to previous reports (Tepper *et al.*, 1989; Golumbek *et al.*, 1991), IL-4 secretion did not affect the growth rate of FS29 cells in athymic mice (data not shown). IL-4 secretion, by murine plasmacytoma, adenocarcinoma or renal cell carcinoma cells had previously been reported to induce an eosinophilic infiltrate *in vivo* (Tepper *et al.*, 1989; Golumbek *et al.*, 1991), and anti-IL-5 antibodies had been shown to partially restore the tumorigenicity of IL-4-secreting cells (Tepper *et al.*, 1992). Therefore, we monitored the effect of secretion of IL-5, the major growth factor for cells of the eosinophil lineage, upon FS29 tumour formation. Figure 1 shows that IL-5 secretion did not cause any slowing of tumour growth in syngeneic animals; no eosinophil infiltrate was observed in IL-5-secreting tumours (data not shown). To observe the effect of simultaneous IL-2 and IL-4 secretion, a doubly-transfected clone secreting sub-optimal levels of both cytokines was isolated. Additional secretion of IL-2, by the non-rejecting IL-4 secreting clone IL-4.1, was able to induce tumour rejection (Table I). This co-operative effect suggested that the two cytokines might act by mobilisation of different effector mechanisms.

### *Host cells infiltrating cytokine-secreting FS29 tumours*

To examine the host cells involved in the response to cytokine-secreting FS29 cells, small tumour nodules were excised and infiltrating host cells were examined morphologically and by immunohistochemical staining. While few host cells were present in the unmodified FS29 tumour nodule, a pronounced lymphocytic infiltrate was present in

**Figure 2** Histopathology of cytokine secreting tumours. Tumour nodules were excised 7 to 10 days after injection of: $10^6$ FS29Neo in C57bl mice (**a**), FS29IL-2.1 in C57bl mice (**b**), FS29IL-4.2 in C57bl mice (**c**), FS29IL-4.2 in nu/nu mice (**d**). Sections were stained with haematoxylin and eosin and photographed at a magnification of 500 ×.

**Table II** Characterisation of host cell infiltrate in FS29 tumour nodules

| | *FS29Neo* | *FS29IL2.1* | *FS29IL4.2* |
|---|---|---|---|
| *Haematoxylin and eosin* | | | |
| PMN | +/− | ++ | +++ |
| macrophages | +/− | + | +++ |
| lymphocytes | +/− | ++++ | + |
| *Immunohistochemistry* | | | |
| anti-CD4 | +/− | + | + |
| anti-CD8 | +/− | ++++ | + |
| anti-B cell | − | + | + |
| anti-IL2R | − | ++ | + |
| anti-IL4R | − | + | +++ |

A summary of morphological and immunohistochemical analysis of tumour nodules, explanted between 7 and 14 days after injection of $10^6$ FS29Neo, FS29IL-2.1 and FS29IL-4.2 cells in C57bl mice.

the IL-2-secreting tumours (Figure 2). The majority of these cells were CD8 +, with some CD4 + cells and B cells (Table II). In contrast, the IL-4-secreting tumours were characterised by a macrophage and granulocyte infiltrate, with a small number of CD8 + and CD4 + lymphocytes (Figure 2, Table II). This inflammatory cell infiltrate was not observed in IL-4-secreting tumour nodules from athymic animals (Figure 2) and thus appears to be dependent on a T lymphocyte response.

*Cytokine-secreting cells are selectively lost from tumours*

To assess the potential of cytokine gene delivery to existing tumours *in vivo* as a tumour therapy, the ability of cytokine-secreting FS29 cells to induce rejection of admixed, unmodified cells was measured. Figure 3 shows that neither IL-2, nor IL-4-secreting FS29 cells caused any slowing of growth of an equal number of admixed non-secreting FS29 cells. When the IL-2, or IL-4-secreting cells were present in a 10-fold excess, initial delay in the growth of the unmodified cells was observed, but rapidly growing tumours eventually formed in all animals (Figure 3). These data are in contrast to several previous studies, which have demonstrated effective induction of parental tumour rejection by admixture of 50% IL-2 (Gansbacher *et al.*, 1990; Ley *et al.*, 1991), or IL-4-secreting cells (Tepper *et al.*, 1989; Golumbek *et al.*, 1991).

To investigate the reason for this lack of effect of cytokine secretion on adjacent unmodified FS29 cells, tumour nodules were explanted soon after injection of IL-2 and parental cell admixtures. When the level of IL-2 secretion from such explanted cells was analysed, admixtures of IL-2-secreting and parental cells were found still to secrete IL-2 after 7 days *in vivo* growth (Figure 4). However, following 14 days of tumour development, IL-2 secretion was lost (Figure 4). This correlated with loss of IL-2-encoding retroviral DNA sequences, which could be detected after 7 days but not 14 days tumour growth, in the explanted cells (Figure 5, upper panel, tracks 5 and 6). Similar analysis of admixtures of IL-4-secreting and parental cells demonstrated greatly reduced IL-4 secretion after 14 days growth *in vivo* (Figure 4) and undetectable IL-4-encoding retroviral DNA (Figure 5, lower panel, track 8). A more rapid loss of IL-2-secreting cells could be clearly observed in an admixture of IL-2 and IL-4-secreting cells. IL-2 and IL-4 rates of secretion by tumours explanted after 7 days were similar to the injected cells (Figure 4); both IL-2 and IL-4 retroviral DNA could be detected (Figure 5, upper panel, track 7 and lower panel, track 9). However, following 14 days growth of the admixture IL-2 secretion (Figure 4) and retroviral DNA sequence (Figure 5, upper panel, track 8) were reduced, while IL-4 secretion (Figure 4) and retroviral DNA (Figure 5, lower panel, track 11) were maintained. These data show that a strong selective pressure against IL-2-secreting FS29 cells and a slightly weaker selective pressure against IL-4-secreting FS29 cells, results in their loss from cell admixtures. This

**Figure 3** Growth of admixtures of cytokine secreting and non-secreting tumour cells. FS29IL-2.1 cells (**a**) or FS29IL-4.1 cells (**b**) were mixed with FS29Neo at ratios of 1:1 ($10^6$ cytokine secretors + $10^6$ FS29Neo), and 10:1 ($10^6$ cytokine secretors + $10^5$ FS29Neo), and injected into each flank of C57bl mice. As controls, mice were injected with $10^5$ or $10^6$ FS29Neo cells.

provides an explanation for the inability of such cytokine secreting FS29 cells to induce rejection of admixed parental cells. These data suggest that direct retroviral-mediated cytokine gene delivery to tumour cells *in vivo* will not be able to induce tumour rejection, even if delivery to over 50% of cells within a tumour were feasible.

*IL-4-secreting cells can protect animals from parental tumour challenge.*

An alternative application of retroviral cytokine gene delivery in cancer therapy would be the use of tumour cells, cultured following primary lesion excision and infected with cytokine-encoding retroviruses, as an injection to enhance eradication of minimal residual disease. The efficacies of

**Figure 4** Cytokine secretion of explanted tumours. C57bl mice were injected with either $10^6$ FS29IL-2.1, $10^6$ FS29IL-4.2, $10^6$ FS29IL-2.1 + $10^6$ FS29Neo, $10^6$ FS29IL-4.2 + $10^6$ FS29Neo, or $10^6$ FS29IL-2.1 + $10^6$ FS29IL-4.2 into each flank. Tumour nodules were excised at 7 and 14 days and supernatant from cells passaged *in vitro* was assayed for IL-2 (**a**) and IL-4 (**b**) activity.

**Figure 5** Southern blot of explanted tumours. Ten μg of SacI digested genomic DNA, or 10 pg Bam H1 digested plasmid was electrophoresed on an 0.8% agarose gel, transferred to nitrocellulose and hybridised with labelled human IL-2 (upper panel) or murine IL-4 (lower panel) cDNAs. **a**: pZipSVIL-2 plasmid (1) giving the 5 kbp proviral integrant size, FS29Neo (2), FS29IL-2.1 in nu/nu mice, day 7 (3), FS29IL-2.1 day 70 (4), FS29Neo + FS29IL-2.1 day 7 (5), FS29Neo + FS29IL-2.1 day 14 (6), FS29IL-2.1 + FS29IL-4.2 day 7 (7), FS29IL-2.1 + FS29IL-4.2 day 14 (8). **b**: pZipSVIL-4 plasmid (1) showing the 4.1 kbp and 0.9 kbp proviral integrant bands, FS29Neo (2) showing genomic IL-4 bands of 6.2, 4.6, 4.0, 2.2, and 0.75 kbp, FS29IL-4.2 in nu/nu mice, day 7 (3), FS29IL-4.2 day 7 (4), FS29IL-4.2 day 32 (5), FS29IL-4.2 + FS29Neo day 7 (6), FS29IL-4.2 + FS29Neo day 11 (7), FS29IL-4.2 + FS29Neo day 14 (8), FS29IL-4.2 + FS29IL-2.1 day 7 (9), FS29IL-4.2 + FS29IL-2.1 day 11 (10), FS29IL-4.2 + FS29IL-2.1 day 14 (11).

parental, IL-2 and IL-4-secreting FS29 cells in the induction of lasting protection of syngeneic mice against FS29 tumour challenge were therefore compared. Figure 6 shows that animals which had rejected the IL-4-secreting FS29 cell primary tumour showed considerably delayed tumour incidence, when challenged after 48 days with FS29Neo cells. Five out of 13 of these animals survived tumour-free for over 30 days. In contrast, parental cell injection afforded no lasting protection, confirming the weak basal immunogenicity of this tumour (Figure 6). IL-2-secreting tumour cells also did not protect animals against delayed parental cell challenge (Figure 6). These data suggest that IL-4-secreting tumour cells, while unable to induce a rapid enough immune response to cause the rejection of admixed parental cells, can stimulate an effective long-term anti-tumour response.

### Discussion

This study represents a detailed comparison of the effects of IL-2 and IL-4 secreted by the same tumour. Secretion of a sufficiently high level of IL-2 by FS29 cells resulted in tumour rejection, whereas lower levels of secretion slowed initial growth but resulted in the later appearance of rapidly growing tumours. This could be explained by the loss of the IL-2 transgene and thus IL-2 secretion by these outgrowing cells, which suggests that a strong selective pressure against IL-2 secretion occurred in syngeneic animals. Loss of cytokine was observed with tumour cells secreting either human, or murine IL-2. A similar selection against IL-2 secretion was previously observed in the rat tumour HSN when passaged in syngeneic immunocompetent, but not athymic, animals (Russell *et al.*, 1991). In contrast, IL-4 secretion by FS29 cells resulted in either tumour rejection or the appearance of slow growing tumours which retained the IL-4 transgene and secreted IL-4. Thus, IL-4 secretion was not subject to such a strong immune selection.

However, when either IL-2- or IL-4-secreting tumour cells were admixed with unmodified tumour cells, the parental tumours rapidly emerged. By analysing explanted tumour nodules, it was determined that the IL-2-secreting cells were lost from such admixtures after 14 days, and that IL-4-secreting cells were greatly reduced in proportion at this time. Thus, a weak immune selection against IL-4-secreting cells does occur when they are admixed with unmodified cells. This lack of rejection of admixed, unmodified cells is in contrast to previous studies with other IL-2-secreting (Gansbacher *et al.*, 1990; Ley *et al.*, 1991) or IL-4-secreting (Golumbek *et al.*, 1991; Tepper *et al.*, 1989) tumour cells. The level of cytokine secretion that we have achieved is similar, or higher than that reported in these studies; perhaps the difference can be attributed to a lower intrinsic immunogenicity of the FS29 tumour. The strong selection of cytokine secreting cells from an admixture implies specific, local immune stimulation by such cells. Furthermore, these data would argue against direct cytokine gene delivery to tumours *in situ* as an effective therapy, with the efficiency of currently available gene therapy techniques.

A more feasible therapeutic approach appears to be the injection of cytokine secreting tumour cells. Animals which had rejected IL-4-secreting FS29 cells were protected against parental tumour when challenged after 48 days. IL-2-

**Figure 6** Growth of secondary challenge tumours. Mice were inoculated with $10^6$ FS29Neo, FS29IL-2.1 or FS29IL-4.2 cells. Any tumours growing at 18 days were excised; all surviving mice, and a control group, were rechallenged after 48 days, on the opposite flank, with $10^6$ FS29Neo and tumour growth monitored. Animals with tumour of greater than 0.4 cm which was increasing in size were considered tumour positive. Percentage of tumour free mice is shown as a function of time. *$P < 0.05$ when tumour incidence compared with naive controls on day 30 using a two-tailed Fisher's exact probability test.

secreting, or parental tumour injected animals showed no protection after this period. From analysis of the host cell infiltrate in tumour nodules undergoing rejection, IL-4 and IL-2 were clearly stimulating different subsets of cells. IL-2 recruited largely CD8 + lymphocytes, whereas IL-4 stimulated macrophages, granulocytes, and some lymphocytes. Indeed, secretion of both IL-2 and IL-4 by the same cell led to enhanced tumour rejection. The stimulation of an inflammatory infiltrate by IL-4 has previously been reported in a murine plasmocytoma (Tepper *et al.*, 1989), murine adenocarcinoma (Tepper *et al.*, 1989) and murine renal cell carcinoma (Golumbek *et al.*, 1991). The importance of such an infiltrate has been demonstrated by the inhibition of tumour rejection in the presence of anti-granulocyte antibodies (Tepper *et al.*, 1992). However in previous studies IL-4 slowed tumour growth, or led to tumour rejection in immunodeficient animals where a similar infiltrate was observed (Tepper *et al.*, 1989; Tepper *et al.*, 1992). As a variety of cell types express receptors for IL-4 (Paul, 1991), a direct effect of this cytokine on the growth of some tumours may be possible. The effects of IL-4 in the FS29 model depend on the presence of T lymphocytes as we did not observe any slowing of tumour growth, or cell infiltrate, in athymic animals. Thus, IL-4 stimulation of T cells recruits inflammatory cells, perhaps by the stimulation of further cytokine production.

While induction of protective immunity clearly requires T cell stimulation, the inflammatory cell infiltrate may also be crucial in the generation of an optimal response. A previous study has demonstrated that IL-4-secreting renal carcinoma cells protect against subsequent tumour challenge and can cure animals pre-injected with a small number of parental cells (Golumbek *et al.*, 1991). However, another report describes a complete absence of induction of protection by IL-4-secreting plasmacytoma cells (Tepper *et al.*, 1992). Two reports described lasting protection induced by IL-2-secreting cells (Gansbacher *et al.*, 1990; Ley *et al.*, 1991), the work of Ley *et al.* (Ley *et al.*, 1991) demonstrated an enhanced protection compared to parental mastocytoma cells. The study of Fearon *et al.* described short-term protection induced by IL-2-secreting colon tumour cells which is greatly diminished after 28 days (Fearon *et al.*, 1990). Such differences observed in previous studies might be ascribed to differential intrinsic immunogenicity of the various tumours. Our direct comparison, in the FS29 sarcoma model, suggests that IL-4-secreting cells provide the better protection. The greater protective response induced by IL-4 may be partly explained by two of our observations. Firstly, IL-4 recruits different subsets of host immune cells, as demonstrated by the different infiltrate observed in IL-4 compared with IL-2-secreting tumours. Some of the cells recruited by IL-4, but not by IL-2, may be important to the establishment of a long-term anti-tumour response. Secondly, as IL-4-secreting cells suffer a less stringent host immune selection, IL-4-secreting tumour cells are maintained for longer than their IL-2 counterparts. This longer period of immune stimulation may also be important in the generation of a greater response.

We would like to thank Janine Salter for immunohistochemical analysis. pZipNeoSV(X) was provided by Prof. R.C. Mulligan, pZipNeoSVIL-2 by Prof. T. Taniguchi, a murine IL-2 cDNA by DNAX (Palo Alto) and a murine IL-5 cDNA by Dr C. Sanderson (NIMR, London) to whom we are also indebted for performing the IL-5 bioassays. The anti murine IL-4 receptor antibody M1 was a gift from Immunex (Seattle) and the anti murine IL-2 receptor antibody 7D4 from Prof. T. Malek.

This work was supported by the Cancer Research Campaign. I.A.M. acknowledges the support of the Restoration of Appearance and Function Trust (RAFT) and the Emmandjay Trust.

Abbreviations: IL-2, interleukin 2; IL-4, interleukin-4; IL-5, interleukin 5; γ-IFN, gamma interferon; TNF-α, tumour necrosis factor alpha.

### References

ASHER, A., MULE, J., KASID, A., RESTIFO, N., SALO, J., REICHERT, C., JAFFE, G., FENDLY, B., KRIEGLER, M. & ROSENBERG, S. (1991). Murine tumour cells transduced with the gene for tumor necrosis factor-α. *J. Immunol.*, **146**, 3227–3234.

BLANKENSTEIN, T., QIN, Z., UBERLA, K., MULLER, W., ROSEN, H., VOLK, H.-D. & DIAMANTSTEIN, T. (1991). Tumour suppression after tumor cell-targeted tumor necrosis factor α gene transfer. *J. Exp. Med.*, **173**, 1047–1052.

BOHLE, W., SCHLAG, P., LIEBRICH, W., HOHENBERGER, P., MANASTERSKI, M., MOLLER, P. & SCHIRRMACHER, V. (1990). Postoperative active specific immunisation in colorectal cancer patients with virus-modified autologous tumour-cell vaccine. *Cancer*, **66**, 1517–1523.

BOON, T., SNICK, J.V., PEL, A.V., UYTTENHOVE, C. & MARCHAND, M. (1980). Immunogenic variants obtained by mutagenesis of mouse mastocytoma P815. II. T lymphocyte-mediated cytolysis. *J. Exp. Med.*, **152**, 1184–1193.

BOONE, C. & BLACKMAN, K. (1972). Augmented immunogenicity of tumour cell homogenates infected with influenza virus. *Cancer Res.*, **32**, 1018–1022.

BRUNNER, K., MACDONALD, R. & CEROTTINI, J. (1981). Quantitation and clonal isolation of cytolytic T lymphocyte precursors selectively infiltrating murine sarcoma virus-induced tumors. *J. Exp. Med.*, **154**, 362–373.

CAMPBELL, H., TUCKER, W., HORT, Y., MARTINSON, M., MAYO, G., CLUTTERBUCK, E., SANDERSON, C. & YOUNG, I. (1987). Molecular cloning, nucleotide sequence, and expression of the gene encoding human eosinophil differentiation factor (interleukin 5). *Proc. Natl Acad. Sci. USA*, **84**, 6629–6633.

CEPKO, C., ROBERTS, B. & MULLIGAN, R. (1984). Construction and applications of a highly transmissible murine retrovirus shuttle vector. *Cell*, 1053–1062.

COLOMBO, M., FERRARI, G., STOPPACCIARO, A., PARENZA, M., RODOLFO, M., MAVILIO, F. & PARMIANI, G. (1991). Granulocyte colony-stimulating factor gene transfer suppresses tumorogenicity of a murine adenocarcinoma *in vivo*. *J. Exp. Med.*, **173**, 889-897.

DANOS, O. (1991). Construction of retroviral packaging cell lines. *Practical Molecular Virology*. Humana. 1, ed. 17-28.

ECCLES, S., HECKFORD, S. & ALEXANDER, P. (1980). Effect of cyclosporin A on the growth and spontaneous metastasis of syngeneic animal tumours. *Br. J. Cancer*, **42**, 252-259.

ESUMI, N., HUNT, B., ITAYA, T. & FROST, P. (1991). Reduced tumourogenicity of murine tumour cells secreting γ-Interferon is due to nonspecific host responses and is unrelated to class I major histocompatibility complex expression. *Cancer Res.*, **51**, 1185-1189.

FEARON, E., ITAYA, T., HUNT, B., VOGELSTEIN, B. & FROST, P. (1988). Induction in a murine tumor of immunogenic tumor variants by transfection with a foreign gene. *Cancer Res.*, **48**, 2975-2980.

FEARON, E., PARDOLL, D., ITAYA, T., GOLUMBEK, P., KARASUYAMA, H., VOGELSTEIN, B. & FROST, P. (1990). Interleukin-2 production by tumour cells bypasses T helper function in the generation of an antitumor response. *Cell*, **60**, 397-403.

GANSBACHER, B., BANNERJI, R., DANIELS, B., ZIER, K., CRONIN, K. & GILBOA, E. (1990). Retroviral vector-mediated γ-Interferon gene transfer into tumor cells generates potent and long lasting antitumor immunity. *Cancer Res.*, **50**, 7820-7825.

GANSBACHER, B., ZIER, K., DANIELS, B., CRONIN, K., BANNERJI, R. & GILBOA, E. (1990). Interleukin 2 gene transfer into tumour cells abrogates tumorogenicity and induces protective immunity. *J. Exp. Med.*, **172**, 1217-1224.

GILLIS, S. & SMITH, K. (1977). Long term culture of tumour-specific cytotoxic T cells. *Nature*, **268**, 154-156.

GOLUMBEK, P., LAZENBY, A., LEVITSKY, H., JAFFEE, L., KARASUYAMA, H., BAKER, M. & PARDOLL, D. (1991). Treatment of established renal cancer by tumor cells engineered to secrete Interleukin-4. *Science*, **254**, 713-717.

HOCK, H., DORSCH, M., DIAMANTSTEIN, T. & BLANKENSTEIN, T. (1991). Interleukin 7 induces CD4+ T cell-dependent tumor rejection. *J. Exp. Med.*, **174**, 1291-1298.

HUI, K., GROSVELD, F. & FESTENSTEIN, H. (1984). Rejection of transplantable AKR leukaemia cells following MHC DNA-mediated cell transformation. *Nature*, **311**, 750-752.

ITO, T., WANG, D.-Q., MARU, M., NAKAJIMA, K., KATO, S., KURIMURA, T. & WAKAMIYA, N. (1990). Antitumor efficacy of vaccinia virus-modified tumor cell vaccine. *Cancer Res.*, **50**, 6915-6918.

KLEIN, G., SJOGREN, H., KLEIN, E. & HELLSTROM, K. (1960). Demonstration of resistance against methylcholanthrene-induced sarcomas in the primary autochthonous host. *Cancer Res.*, **20**, 1561-1572.

KOBAYASHI, H., SENDO, F., SHIRAI, T., KAJI, H., KODAMA, T. & SAITO, H. (1969). Modification in growth of transplantable rat tumours exposed to Friend virus. *J. Natl Cancer Inst.*, **42**, 413-419.

LEE, F., YOKOTA, T., OTSUKA, T., MEYERSON, P., VILLARET, D., COFFMAN, R., MOSMANN, T., RENNICK, D., ROEHM, N., SMITH, C., ZLOTNIK, A. & ARAI, K. (1986). Isolation and characterisation of a mouse interleukin cDNA clone that expreses B-cell stimulatory factor 1 and T-cell and mast-cell-stimulating activities. *Proc. Natl Acad. Sci. USA*, **83**, 2061-2065.

LEY, V., LANGLADE-DEMOYEN, P., KOURILSKY, P. & LARSSON-SCIARD, E. (1991). Interleukin 2-dependent activation of tumor-specific cytotoxic T lymphocytes *in vivo*. *Eur. J. Immunol.*, **21**, 851-854.

LICHTMAN, A., KURT-JONES, E. & ABBAS, A. (1987). B cell stimulatory factor 1 and not interleukin 2 is the autocrine growth factor for some helper T lymphocytes. *Proc. Natl Acad. Sci. USA*, **84**, 824.

LINDENMANN, J. & KLEIN, P. (1967). Viral oncolysis: increased immunogenicity of host cell antigen associated with influenza virus. *J. Exp. Med.*, **126**, 93-108.

MARKOWITZ, D., GOFF, S. & BANK, A. (1988). Construction and use of a safe and efficient amphotropic packaging cell line. *Virology*, **167**, 400-406.

MCBRIDE, W., THACKER, J., COMORA, S., ECONOMOU, J., KELLEY, D., HOGGE, D., DUBINETT, S. & DOUGHERTY, G. (1992). Genetic modification of a murine fibrosarcoma to produce interleukin 7 stimulates host cell infiltration and tumour immunity. *Cancer Res.*, **52**, 3931-3937.

MILLER, A. (1992). Human gene therapy comes of age. *Nature*, **375**, 455-460.

MILLER, A. & BUTTIMORE, C. (1986). Redesign of retrovirus packaging cell lines to avoid recombination leading to helper virus production. *Mol. & Cell. Biol.*, **6**, 2895-2902.

OHARA, J. & PAUL, W. (1985). B cell stimulatory factor BSF-1: production of a monoclonal antibody and molecular characterisation. *Nature*, **315**, 333.

PAUL, W. (1991). Interleukin-4: a prototypic immunoregulatory lymphokine. *Blood*, **77**, 1859-1870.

PREHN, R. & MAIN, J. (1957). Immunity to methyl-cholanthrene-induced sarcomas. *J. Natl Cancer Inst.*, **18**, 769-778.

ROSENBERG, S. (1992*a*). Immunisation of cancer patients using autologous cancer cells modified by insertion of the gene for Interleukin-2. *Human Gene Therapy*, **3**, 75-90.

ROSENBERG, S. (1992*b*). Immunisation of cancer patients using autologous cancer cells modified by insertion of the gene for tumour necrosis factor. *Human Gene Therapy*, **3**, 57-73.

ROSENBERG, S., LOTZE, M., YANG, J., AEBERSOLD, P., LINEHAN, W., SEIPP, C. & WHITE, D. (1989). Experience with the use of high-dose interleukin-2 in the treatment of 652 cancer patients. *Ann. Surg.*, **210**, 474-485.

RUSSELL, S., ECCLES, S., FLEMMING, C., JOHNSON, C. & COLLINS, M. (1991). Decreased tumorigenicity of a transplantable rat sarcoma following transfer and expression of an IL-2 cDNA. *Int. J. Cancer*, **47**, 244-251.

SMITH, K. (1988). Interleukin-2: inception, impact, and implications. *Science*, **240**, 1169-1176.

STRATH, M., WARREN, D. & SANDERSON, C. (1985). Detection of eosinophils using an eosinophil peroxidase assay. *J. Immunol. Meth.*, **83**, 209.

SUGIURA, C., ITAYA, T., KONDOH, N., OIKAWA, T., KUZUMAKI, N., TAKEICHI, N., HOSOKAWA, M. & KOBAYASHI, H. (1988). Xenogenization of tumor cells by transfection with plasmid containing *env* gene of Friend leukaemia virus. *Jpn. J. Cancer Res.*, **79**, 1259-1263.

TANAKA, K., ISSELBACHER, K., KHOURY, G. & JAY, G. (1985). Reversal of oncogenesis by the expression of a major histocompatibility complex class I gene. *Science*, **228**, 26-30.

TEPPER, R., COFFMAN, R. & LEDER, P. (1992). An eosinophil-dependent mechanism for the antitumour effect of interleukin-4. *Science*, **257**, 548-551.

TEPPER, R., PATTENGALE, P. & LEDER, P. (1989). Murine Interleukin-4 displays potent anti-tumor activity *in vivo*. *Cell*, **57**, 503-512.

UYTTENHOVE, C., MARYANSKI, J. & BOON, T. (1983). Escape of mouse mastocytoma P815 after nearly complete rejection is due to antigen-loss variants rather than immunosuppression. *J. Exp. Med.*, **157**, 1040-1052.

WALLICH, R., BULBUC, N., HAMMERLING, G., KATZAV, S., SEGAL, S. & FELDMAN, M. (1985). Abrogation of metastatic properties of tumor cells by de novo expression of H-2K antigens following H-2 gene transfection. *Nature*, **315**, 301-305.

WATANABE, Y., KURIBAYASHI, K., MIYATAKE, S., NISHIHARA, K., NAKAYAMA, E.-I., TANIYAMA, T. & SAKATA, T.-A. (1989). Exogenous expression of mouse interferon γ-cDNA in mouse neuroblastoma C1300 cells results in reduced tumorigenicity by augmented anti-tumor immunity. *Proc. Natl Acad. Sci. USA*, **86**, 9456-9460.

YAMADA, G., KITAMURA, Y., SONODA, H., HARADA, H., TAKI, S., MULLIGAN, R., OSAWA, H., DIAMANSTEIN, T., YOKOYAMA, S. & TANIGUCHI, T. (1987). Retroviral expression of the human IL-2 gene in a murine T cell line results in cell growth autonomy and tumorigenicity. *EMBO J.*, **6**, 2705-2709.